# 2-Kidney-1-clip hypertension is not attenuated in mice lacking the transient receptor potential vanilloid type 1 (TRPV1) channel

**DOI:** 10.3389/fphys.2025.1713864

**Published:** 2025-11-28

**Authors:** Sean D. Stocker, Isabella Benoit, Jacob B. Sullivan, Caroline B. Ferreira

**Affiliations:** Department of Neurobiology, University of Pittsburgh School of Medicine, Pittsburgh, PA, United States

**Keywords:** autonomic nervous system, blood pressure, hypertension, dorsal root ganglion, kidney, renovascular

## Abstract

**Introduction:**

Chemical ablation of renal sensory nerves using agonists for transient receptor potential vanilloid-1 (TRPV1) lowers arterial blood pressure (ABP) in multiple experimental models of hypertension. Interestingly, both afferent renal nerve activity and arterial blood pressure were significantly attenuated in male Trpv1^−/−^ rats after 2-kidney-1-clip (2K1C) renovascular hypertension. However, TRPV1 expression in sensory neurons differs across species and is lower in mice versus rats or humans. Therefore, the current study assessed the proportion of TRPV1 in mouse renal sensory neurons and tested whether deletion of TRPV1 altered renovascular hypertension in mice.

**Methods:**

2K1C surgery was performed by placement of a 0.5mm length of polyetrafluoroethylene tubing around the right renal artery. Experiment 1 quantified the proportion of TRPV1 mouse renal sensory neurons in both sham and 2K1C after a kidney injection of the tracer wheat germ agglutinin conjugated to AlexaFluor 647. Experiment 2 assessed ABP using telemetry in WT and Trpv1^−/−^ mice after 2K1C.

**Results:**

First, the majority of retrogradely labeled neurons were located in the ipsilateral T10-L2 dorsal root ganglion and small to medium sized (10-29um diameter). Approximately 60% were TRPV1-positive. Second, 2K1C significantly increased ABP in both male and female WT and Trpv1^−/−^ mice. However, the magnitude of the hypertension was not statistically different between strain and sex. Depressor responses to ganglionic blockade also did not differ between strains and sex.

**Conclusion:**

These findings suggest that a subset of renal sensory neurons in the mouse are TRPV1-positive, and renovascular 2K1C hypertension is not attenuated in the Trpv1^−/−^ mouse.

## 1 Introduction

Renal sensory nerves contribute to multiple experimental forms of hypertension and chronic kidney disease (Osborn and Foss, 2017; Osborn et al., 2021). The peripheral afferent nerve endings innervate several cell types within the kidney including the vasculature, tubules, glomeruli and pelvic wall (Ferguson and Bell, 1988; Ferguson et al., 1988; Marfurt and Echtenkamp, 1991; Kopp, 2015; Tyshynsky et al., 2023). These fibers are activated by changes in renal perfusion pressure or ischemia, local metabolic factors or chemicals, and alterations in pelvic pressure and chemical composition of urine (Recordati et al., 1981; Recordati et al., 1980; Recordati et al., 1978; Katholi et al., 1983; DeLalio and Stocker, 2021). Neurophysiological recordings demonstrate afferent renal nerve activity (ARNA) is elevated in rodent models of deoxcorticosterone-salt and 2-kidney-1-clip (2K1C) renovascular hypertension (Banek et al., 2016; Ong et al., 2019). Dorsal rhizotomy lowered arterial blood pressure (ABP) in rats with chronic renal failure or 2K1C renovascular hypertension (Wyss et al., 1986; Campese and Kogosov, 1995). More recently, selective chemical ablation of renal sensory fibers by periaxonal application of the transient receptor potential vanilloid type 1 (TRPV1) agonist capsaicin reduced sympathoexcitation and lowered ABP in deoxycorticosterone-salt, 2K1C, and models of chronic kidney disease (Ong et al., 2019; Foss et al., 2015; Lopes et al., 2020; Lauar et al., 2023).

Although chemical ablation of sensory fibers using TRPV1 agonists destroy TRPV1-expressing neurons, this approach does not distinguish between the TRPV1 channel versus TRPV1-expressing fibers as these sensory neurons express additional receptor and/or channels. TRPV1 channels are widely expressed in sensory neurons and respond to noxious and mechanical stimuli, pH, and local chemical factors (Julius, 2013; Nilius, 2007). We recently reported that the majority (∼85%) of renal sensory neurons in the rat are TRPV1-positive, and deletion of TRPV1 using Trpv1^−/−^ rat reduced sympathetic nerve activity and ABP in 2K1C renovascular hypertension (Stocker and Sullivan, 2023). However, there may be species differences in TRPV1 expression within sensory neurons. For example, a harmonized atlas of the dorsal root ganglion using single-cell or single-nuc RNAseq data sets noted a higher level of TRPV1 expression in humans and rats versus mice (Bhuiyan et al., 2024). Consistent with this notion, *in vitro* patch-clamp recordings indicate a higher proportion of dorsal root ganglion neurons respond to TRPV1 agonists in rats versus mice (Chakraborty et al., 2016; Sculptoreanu et al., 2008; Chakraborty et al., 2017; Zhang et al., 2007; Leffler et al., 2006; Kistner et al., 2016; Han et al., 2016). Using whole-cell recordings, >70% of rat renal sensory neurons are responsive to capsaicin (Wang et al., 2010). No such data currently exists in the mouse. Given the potential differences in Trpv1 expression between species and especially rodents, the current study assessed the proportion of TRPV1 mouse renal sensory neurons and also tested the contribution of TRPV1 channel in 2K1C hypertension using wild-type (WT) and Trpv1^−/−^ mice.

## 2 Methods

The data that support the findings of this study are available from the corresponding author upon reasonable request.

### 2.1 Animals

All of the experimental procedures conform to the National Institutes of Health Guide for the Care and Use of Laboratory Animals and were approved by the Institutional Animal Care and Use Committee at the University of Pittsburgh School of Medicine. Mice were housed in a temperature-controlled room (22 °C ± 1 °C) with a 12-h reverse light-dark cycle (lights off 10 A M-10PM) and given access to food (LabDiets chow #5P76) and water *ad libitum*. Male and female Trpv1^−/−^ mice (Strain #003770, Jackson Laboratories) were crossed with male or female C56BL/6J (strain #00664, Jackson Laboratories) to generate Trpv1^+/−^ mice. Trpv1^+/−^ were subsequently bred to produce Trpv1^−/−^ and Trpv1^+/+^ littermates for experiments. The Trpv1^−/−^ mouse has been extensively characterized (Caterina et al., 2000).

Genotypes were confirmed using an ear punch and PCR using primers as specified by The Jackson Laboratory (oIMR0297, 5′-CACGAGACTAGTGAGACGTG-3′; oIMR1561, 5′-CCTGCTCAACATGCTCAT TG-3′; oIMR1562, 5′-TCCTCATGCACTTCAGGAAA-3′). In addition, we performed an eye-wipe test to confirm the genotypes as described previously (Stocker and Sullivan, 2023; Taylor et al., 2008). Briefly, the number of eye-wipes were quantified after topical application of capsaicn (100 uM, 20 uL) to the eye over a 2-min period. As previously reported, capsacin evoked a significant eye-wipe response in WT but not Trpv1^−/−^ mice (25 ± 2 versus 0 ± 0 wipes per 2 min; n = 22 and 17 respectively, P < 0.001).

2K1C surgery was performed as described previously (Ong et al., 2019; Warner et al., 2012; Kashyap et al., 2016). Mice were anesthetized with 2% isoflurane (in 100% O_2_). Through a retroperitoneal incision, the right kidney was gently retracted. A small section of the renal artery at the midpoint between the aorta and kidney was isolated free from the renal vein and/or renal nerves. A 0.5-mm length polytetrafluoroethylene (PTFE) tubing (inner diameter 0.008 in. × outer diameter 0.014 in., item no. SUBL 140; Braintree Scientific) was cut longitudinally, placed around the renal artery, and secured by two 10–0 circumferential sutures. The incisions were closed with suture and the animals treated with Ethiqa (3.25 mg/kg, sc) and enrofloxacin (2 mg/kg, sc).

### 2.2 Experiment 1: quantification of TRPV1 neurons in mouse renal sensory neurons

To determine the proportion of renal sensory neurons that express TRPV1, male C57BL/6J mice (8–10 weeks of age, Strain #00664, Jackson Laboratories) were anesthetized with isoflurane (2%–3% in 100% O_2_), and 2K1C or sham surgery was performed as described above. At 18–21 days later, mice were re-anesthetized with isoflurane (2%–3% in 100% O_2_). Through a retroperitoneal incision, the right kidney was exposed and gently retracted to visualize the renal hilum. A glass micropipette (outer diameter 20–40 µm) containing wheat germ agglutinin conjugated to AlexaFluor 647 (WGA-647, 5%; ThermoFisher) was lowered into the kidney using coordinates relative to the renal hilum: 1.5–2.0 mm lateral and 1.5–1.6 mm ventral to the surface. Three injections (500 nL each) were performed separated by 1.5 mm toward each pole as described previously (Ong et al., 2019). A few drops of KWIK-SIL (World Precision Instruments) were applied to the injection site to prevent tracer leaking from the kidney.

At 5 days later, animals were anesthetized with isoflurane and perfused transcardially with 4% paraformaldehyde (in 10 mM phosphate buffered saline, pH 7.3). The ipsilateral dorsal root ganglia (DRG) at T7–L2 were harvested, postfixed in 4% paraformaldehyde for 1 h at 4 °C, immersed in 30% sucrose overnight, sectioned at 20 µm using a cryostat in 2 sets, and stored at −80 °C until processed. One set contained 10–15 sections per DRG depending on the level. Sections were incubated in a rabbit anti-TRPV1 antibody (1:2,000 at 4 °C for 24 h, Alomone ACC-030) and visualized with donkey anti-rabbit AlexaFluor 555 (1:250 at 4 °C for 24 h, ThermoFisher). To label DRG neurons with an additional control marker, sections were then incubated with Isolectin GS-IB4 Alexa Fluor 488 (1 ug/mL at 4 °C for 24 h, ThermoFisher I121411). Isolectin GS-IB4 is a ligand that binds to nonpeptidergic sensory neurons (Silverman and Kruger, 1990). Prior studies indicate TRPV1 versus IB4 + nonpeptidergic neurons have distinct electrophysiological properties (Stucky and Lewin, 1999). The specificity of the rabbit TRPV1 antibody has been validated previously (Stocker and Sullivan, 2023; Chen et al., 2015). All incubations were performed using 10 mM phosphate buffered saline and 1% Donkey Serum. Sections were coverslipped with Prolong Gold. Labelled cells and kidney injection sites were visualized with a Nikon Ti2 microscope and NIS-Elements software. Cell counts of retrograde labelling, TRPV1 and IB4 immunofluorescence was performed independently by two individuals. Cell diameter was calculated by the average of 6 measurements per cell by measuring the distance from one side of the membrane to the other and extending through the cell nucleus using NIS Elements Software.

### 2.3 Experiment 2: 2K1C hypertension in WT and Trpv1^−/−^ mice

Male and female WT and Trpv1^−/−^ littermates (8–10 weeks of age) were anesthetized with isoflurane (2%–3% in 100% O_2_) and instrumented with PA-C10 telemetry units (Data Sciences) advanced 1.5 cm into the femoral artery with the transmitter body secured subcutaneously in the flank. Mice were treated with Ethiqa and enrofloxacin as described above, returned to home cages, and given 1 week to recover with access to 0.1% NaCl chow (D17020, Research Diets) and distilled water. After a 3-day baseline recording, mice were anesthetized with 2%–3% isoflurane and underwent 2K1C surgery as described above. Ganglionic blockade with chlorisondamine (4 mg/kg, ip) was performed between 1 and 3 p.m. (lights off) on day 0 (before 2K1C surgery) and day 23. The change in ABP was calculated by comparing a 3-min baseline (prior to injection) to a 10-s peak drop. On day 24, mice were anesthetized with isoflurane (3% in 100% oxygen), and the kidneys were harvested and weighed. Telemetry data were analyzed beat to beat for systolic and diastolic ABP. Mean ABP was calculated by diastolic plus one-third of the pulse pressure. Data were analyzed between 12:00 p.m. and 8:00 p.m. (lights off) and 12:00 a.m. and 8:00 a.m. (lights on).

### 2.4 Statistical analysis

Data are presented as mean ± SEM plus individual data points when possible. Data were analyzed using two- or three-way ANOVAs (strain, time, and sex) with repeated measures (Systat 10.2). When significant F values were obtained, a layered Bonferroni paired or independent t-tests were performed to identify differences. If a data set failed normality, a Mann-Whitney U was performed. In all instances, the statistical conclusions were similar between the non-parametric and parametric tests. P < 0.05 was statistically significant for all comparisons. Group sizes are noted in the text and figure legends.

## 3 Results

### 3.1 Experiment 1: quantification of TRPV1 neurons in mouse renal sensory neurons

**FIGURE 1:**
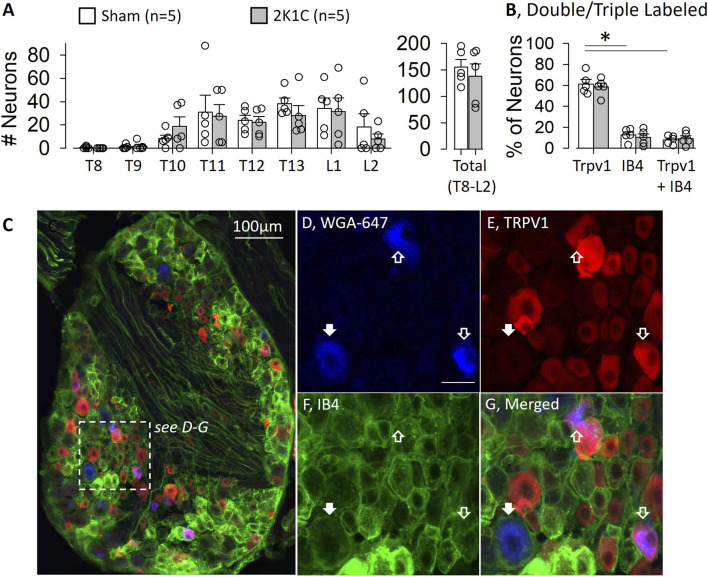
Quantification of Mouse Renal Sensory Neurons Expressing Transient Receptor Potential Vanilliod 1 (TRPV1) Channel. **(A)** Number of retrogradely labelled neurons in the dorsal root ganglion at each thoracic and lumbar spinal segment in both control and 2-kidney-1-clip (2K1C) mice. **(B)** The percentage of retrogradely labelled neurons that were Trpv1, IB4, or Trpv1 plus IB4 positive. Note, there were no statistical differences in the number of renal sensory neurons or percentage of TRPV1, IB4, and TRPV1+IB4 between sham and 2K1C mice. *P < 0.05 Trpv1 vs. IB4 or TRPV1 plus IB4. **(C)** Low power image of mouse dorsal root ganglia visualized for Wheat Germ Agglutinin-647 (WGA-647, blue), TRPV1 (red), and IB4 (green). **(D-G)** Inset images of WGA-647 (blue), TRPV1 (red), IB4 (green), and merged. Unfilled Arrow: TRPV1-positive, IB4- renal sensory neuron; Filled Arrow: TRPV1-negative renal sensory neuron.

The first set of experiments quantified the proportion of mouse renal sensory neurons that express TRPV1 using immunofluorescence. As expected (Ong et al., 2019), the greatest number of renal sensory neurons labeled from the right kidney were located in the T11-L1 DRG (Figure 1A). These numbers did not differ between sham and 2K1C mice. Approximately 60% of mouse renal sensory neurons were TRPV1-positive (sham: 62% ± 4% versus 2K1C: 58% ± 4%, Figure 1B). On the other hand, a significantly smaller proportion were IB4-positive (sham: 13% ± 3% versus 2K1C: 10% ± 3%) or TRPV1+IB4-positive (sham: 8% ± 2% versus 2K1C: 9% ± 3%). Figures 1C–G illustrates an example of a mouse DRG with retrogradely labelled cells from the kidney, TRPV1 immunofluorescence, and IB4 labeling. A subset of renal sensory neurons were TRPV1-positive. As expected, the renal mass was not different between the left and right kidney of sham mice (204 ± 14 mg vs. 191 ± 7mg, respectively); however, 2K1C surgery significantly decreased renal mass of the right versus left kidney (218 ± 14 mg versus 87 ± 16mg, respectively; P < 0.01).

**FIGURE 2:**
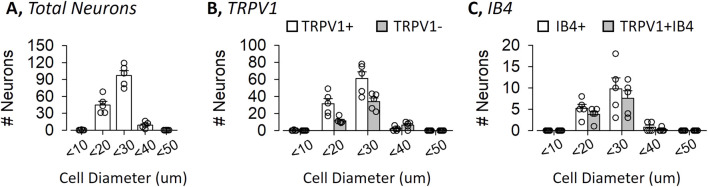
Distribution of Mouse Renal Sensory Neurons as a Function of Cell Diameter Cell diameter was calculated by the average of six measurements per cell and split into 10 um bins. **(A)** Number of renal sensory neurons in the dorsal root ganglion as a function of cell diameter. **(B)** Number of TRPV1-positive versus TRPV1-negative renal sensory neurons. **(C)** Number of IB4+ versus IB4+ plus TRPV1-positive neurons. Values are mean ± SEM and individual data points.

The diameter of most renal sensory neurons were small- (10–19 um, 29% ± 2%) to medium-size (20–29 um, 65% ± 3%) (Figure 2A). TRPV1 neurons had an average diameter of 22.0 ± 0.1 um and were small- (33% ± 4%) or medium-sized (65% ± 4%) (Figure 2B). Similarly, non-TRPV1 renal sensory neurons had an average cell diameter of 24.4 ± 0.2 um and were small- (23% ± 2%) to medium-sized (66% ± 3%). The distribution and size of TRPV1 versus non-TRPV1 neurons did not differ. IB4 or TRPV1 plus IB4 renal sensory neurons had similar diameters (Figure 2C).

### 3.2 Experiment 2A: 2K1C hypertension in male WT and Trpv1^−/−^ mice

**FIGURE 3:**
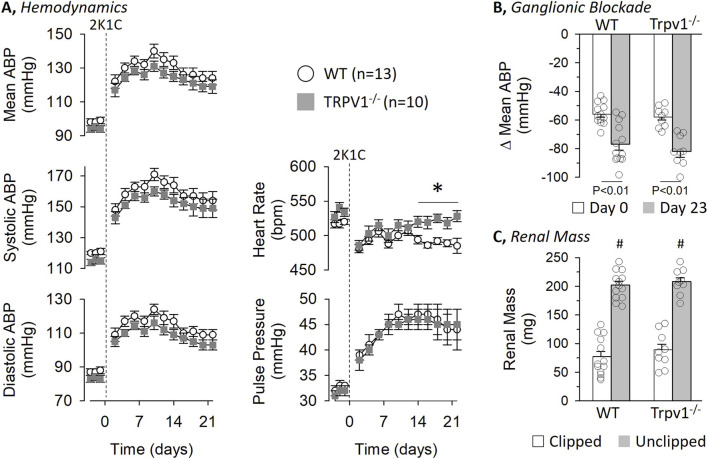
Hemodynamic responses to 2-kidney-1-Clip (2K1C) Hypertension in Wild-Type and Transient Receptor Potential Vanilloid 1 Knockout (Trpv1^−/−^) Male Mice **(A)** Mean ± SEM of mean, systolic, and diastolic ABP as well as pulse pressure and heart rate in wild-type and Trpv1^−/−^ mice. Values were averaged between day and night periods. **(B)** Mean ± SEM and individual data points of the depressor response to ganglionic blockade with chlorisondamine (4 mg/kg, ip) on Day 0 and 23. **(C)** Mean ± SEM and individual data points of kidney mass for both clipped and unclipped kidneys in wild-type and Trpv1^−/−^ mice. *P < 0.05 wild-type vs. Trpv1^−/−^, #P < 0.05 unclipped vs. clipped. Data were analyzed by a 2-way Mixed ANOVA (strain, time) with repeated measures followed by paired or independent t-tests with layered Bonferroni correction when significant F values were obtained.

To test the extent by which TRPV1 channels contribute to renovascular hypertension in mice, 2K1C surgery was performed in WT and Trpv1^−/−^ littermates. Since a recent study in WT and Trpv1^−/−^ rats suggested sex impacted the hemodynamic response to 2K1C hypertension (Stocker and Sullivan, 2023), the data are presented separately for male and female mice. Figure 3 illustrates hemodynamic data before and after 2K1C surgery in male WT and Trpv1^−/−^ mice. 24-h baseline mean, systolic, and diastolic ABP as well as pulse pressure did not differ between strains. 2K1C surgery significantly increased ABP and pulse pressure on Day 2 and remained significantly elevated for the remainder of the study. However, there were no differences in any variable between male WT and Trpv1^−/−^ mice. In addition, baseline heart rate was not different between strains. 2K1C surgery initially decreased heart rate in both strains, and heart rate remained lower in WT versus Trpv1^−/−^ mice between Day 14–22.

Ganglionic blockade with chlorisondamine was performed immediately before and 23 days after 2K1C surgery (Figure 3B). On day 0, chlorisondamine produced a significant fall in mean ABP that did not differ between strains (WT: 113 ± 3 to 57 ± 2 mmHg vs. Trpv1^−/−^: 110 ± 3 to 52 ± 3 mmHg). After 2K1C surgery, the depressor response in both strains was significantly greater at day 23 versus day 0; however, the response was not different between strains (Figure 3B, WT: 140 ± 4 to 63 ± 3 mmHg vs. Trpv1^−/−^: 137 ± 6 to 55 ± 4 mmHg). As expected, unilateral renal stenosis produced a significantly smaller renal mass between the clipped versus unclipped kidney in male WT and Trpv1^−/−^ mice (Figure 3C) but there were not differences between strains. Body weight did not differ between strains at Day 0 (WT: 25.9 ± 0.6 g vs. Trpv1^−/−^: 24.8 ± 0.4 g) or Day 24 (WT: 26.5 ± 0.5 g vs. Trpv1^−/−^: 25.5 ± 0.3 g).

### 3.3 Experiment 2B: 2K1C hypertension in female WT and Trpv1^−/−^ mice

**FIGURE 4:**
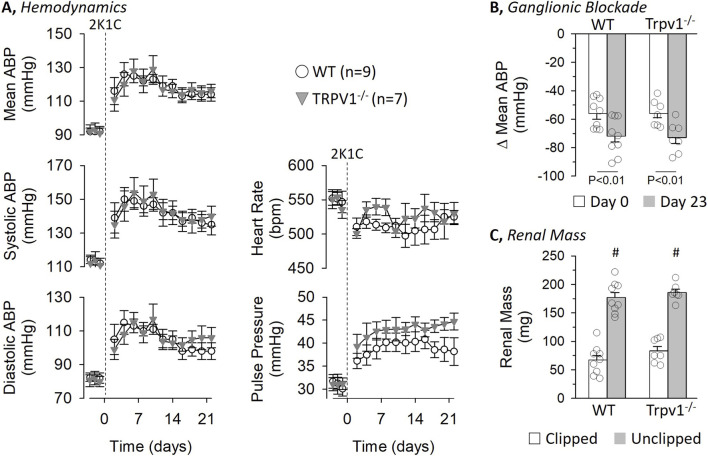
Hemodynamic responses to 2-kidney-1-Clip (2K1C) Hypertension in Wild-Type and Transient Receptor Potential Vanilloid 1 Knockout (Trpv1^−/−^) Female Mice. **(A)** Mean ± SEM of mean, systolic, and diastolic ABP as well as pulse pressure and heart rate in wild-type and Trpv1^−/−^ mice. Values were averaged between day and night periods. **(B)** Mean ± SEM and individual data points of the depressor response to ganglionic blockade with chlorisondamine (4 mg/kg, ip) on Day 0 and 23. **(C)** Mean ± SEM and individual data points of kidney mass for both clipped and unclipped kidneys in wild-type and Trpv1^−/−^ mice. *P < 0.05 wild-type vs. Trpv1^−/−^, #P < 0.05 unclipped vs. clipped. Data were analyzed by a 2-way Mixed ANOVA (strain, time) with repeated measures followed by paired or independent t-tests with layered Bonferroni correction when significant F values were obtained.

A parallel experiment tested the extent by which deletion of TRPV1 channels in female mice altered 2K1C renovascular hypertension. There were no differences in baseline 24-h mean, systolic, and diastolic ABP as well as pulse pressure between WT and Trpv1^−/−^ female mice (Figure 4). 2K1C surgery significantly increased ABP and pulse pressure on Day 2 and remained elevated throughout the remainder of the experiment. However, there were no differences between WT and Trpv1^−/−^ female mice. In addition, baseline heart rate did not differ between female strains. 2K1C surgery produced an initial decreased in heart rate on Day 2 on both strains and remained lower in female WT mice.

Ganglionic blockade with chlorisondamine produced a significant fall in mean ABP on Day 0, and the magnitude of this depressor response did not differ between female WT and Trpv1^−/−^ mice (WT: 114 ± 3 to 58 ± 2 mmHg vs. Trpv1^−/−^: 109 ± 3 to 50 ± 4 mmHg) (Figure 4B). After 2K1C surgery, the depressor response on Day 23 was significantly greater in both strains, but the magnitude was not different between female WT and Trpv1^−/−^ mice (WT: 135 ± 4 to 63 ± 1 mmHg vs. Trpv1^−/−^: 138 ± 5 to 65 ± 5 mmHg). As expected, 2K1C surgery produced a significantly smaller renal mass between the clipped versus unclipped kidney in female WT and Trpv1^−/−^ mice (Figure 4C) but there were no differences between strains. Body weight did not differ between strains at Day 0 (WT: 22.7 ± 1.0 g vs. Trpv1^−/−^: 22.9 ± 1.8 g) or Day 24 (WT: 23.6 ± 0.7 g vs. Trpv1^−/−^: 24.9 ± 0.8 g).

Although data from male and female mice are reported separately (Figures 3, 4), an ANOVA revealed no significant hemodynamic differences between males versus female mice. The only difference revealed was that body weight and mass of unclipped kidneys (contralateral to the stenosis) were significantly larger in male versus female mice regardless of strain.

## 4 Discussion

The present study investigated the proportion of mouse renal sensory neurons that were TRPV1-positive and the extent by which deletion of TRPV1 attenuated 2K1C renovascular hypertension. The majority of mouse renal sensory neurons were small-to-medium-size diameter (10–29um) but only approximately 60% were TRPV1-positive. Second, 2K1C surgery increased ABP in both WT and Trpv1^−/−^ mice, but there were no differences in any hemodynamic variable despite similar changes in renal mass and depressor responses to ganglionic blockade. Collectively, these findings suggest mice have both TRPV1 and non-TRPV1 renal sensory neurons, and TRPV1 channels do not contribute to renovascular hypertension in the mouse.

The current experiments indicate approximately 60% of mouse renal sensory neurons are TRPV1-positive using immunofluorescence. This is a significantly smaller proportion than recently reported for rat renal sensory neurons using the same approach (Stocker and Sullivan, 2023) or whole-cell patch clamp recordings and bath application of capsaicin (Wang et al., 2010). Comparisons across functional studies using *in vitro* patch-clamp recordings support clear species differences with a higher proportion of dorsal root ganglion neurons responsive to the TRPV1 agonist capsaicin in the rat versus mouse (Chakraborty et al., 2016; Sculptoreanu et al., 2008; Chakraborty et al., 2017; Zhang et al., 2007; Leffler et al., 2006; Kistner et al., 2016; Han et al., 2016). This notion is also supported by a recent analysis of RNAseq data sets across multiple species noting TRPV1 expression in dorsal root ganglia was higher in humans and rats versus mice (Bhuiyan et al., 2024). Although these observations suggest potential neurochemical differences between rat and mouse renal sensory neurons, these findings may impact the interpretation of experiments using chemical ablation techniques with TRPV1 agonists. For example, periaxonal application of capsaicin should ablate the majority of renal sensory neurons in the rat, but the same technique should ablate a smaller proportion in the mouse. Although studies using this approach report renal sensory neurons are largely ablated in either species, the conclusion was largely based on assays for calcitonin gene related peptide (Banek et al., 2016; Foss et al., 2015). Peptidergic markers such as calcitonin gene related peptide or substance P highly colocalized in TRPV1 sensory neurons (Cavanaugh et al., 2011), but TRPV1-peptidergic only represent a subset of sensory neurons in the dorsal root ganglion. Thus, assays for peptidergic markers may not fully reflect whether the sensory neuron population is ablated, rather only reflect the presence or absence of TRPV1-peptidergic neurons.

A second major goal of the present study was to assess the extent by which TRPV1 channels contributed to sympathoexcitation and hypertension in a mouse 2K1C renovascular hypertension model. The rationale was based on two observations. First, chemical ablation of renal afferent nerves to the stenosed kidney using high concentrations of the TRPV1 agonist capsaicin reduced ABP at 2–3 weeks after 2K1C surgery in mice (Ong et al., 2019). Similar observations have been reported in rats (Lopes et al., 2020; Lauar et al., 2023). Second, 2K1C elevates ARNA, sympathetic outflow and ABP, and these responses are attenuated in male Trpv1^−/−^ rats (Stocker and Sullivan, 2023). Surprisingly, the current study observed that unilateral renal stenosis increased ABP in both strains and sexes, but there were no differences in any hemodynamic variable between WT and Trpv1^−/−^ mice. These findings mirror a previous study in which DOCA-salt hypertension, a model dependent on renal sensory nerves in the mouse and rat (Foss et al., 2015; Baumann et al., 2024), was also not different between male wild-type and Trpv1^−/−^ mice (Wang et al., 2008). Collectively, these findings indicate 2K1C and DOCA-salt hypertension are not attenuated in the Trpv1^−/−^ mouse.

Several factors may contribute to the differential impact of 2K1C hypertension in the Trpv1^−/−^ rat (Stocker and Sullivan, 2023) versus mouse. Although the proportion of Trpv1-expressing renal sensory neurons differ between the rat and mouse as discussed above, this is unlikely to explain the differential effect on 2K1C hypertension in Trpv1^−/−^. Prior studies indicate that periaxonal application of high concentrations of the TRPV1 agonist lower ABP in both rats and mice after 2K1C (Ong et al., 2019; Lopes et al., 2020; Lauar et al., 2023). Therefore, TRPV1-expressing sensory fibers contribute to 2K1C hypertension in both species; however, the TRPV1 channel does not contribute to 2K1C hypertension in the mouse. A second potential factor may be the degree or severity of hypertension. In the prior study (Stocker and Sullivan, 2023), unilateral stenosis increased mean ABP to ∼150 mmHg in male WT rats but ∼125 mmHg in female WT rats. Deletion of the TRPV1 channel attenuated the hypertension in male rats only. In the present study, 2K1C hypertension was ∼130 mmHg mean ABP and unaffected in Trpv1^−/−^ mice. Further experiments are needed but this raises the possibility that the contribution of the TRPV1 channel could be related to the severity of the hypertension or secondary factors such as end-organ damage (e.g., renal damage).

The premise of the current study was based on observations that chemical ablation of TRPV1-expressing renal sensory fibers lowered ABP in renovascular hypertension. Thus, the working hypothesis is that renal stenosis activates TRPV1-expressing renal sensory fibers (or possibly the TRPV1 channel) to elevate sympathetic outflow and ABP. However, there are several limitations of the present study. Although a prior study in mice reported 2K1C elevated afferent renal nerve activity in mice (Ong et al., 2019), we did not perform afferent renal nerve activity recordings in the current study. Therefore, afferent renal nerve activity may be elevated in both WT and Trpv1^−/−^ mice with 2K1C. This would suggest additional channels or mechanisms activate renal sensory fibers independent of the TRPV1 channel in 2K1C. Second, direct recordings of renal SNA were not performed to assess sympathetic outflow. However, we did perform ganglionic blockade, and these data suggested 2K1C produced sympathoexcitation but there were no differences between strains. Lastly, we utilized Trpv1^−/−^ mice rather than selective deletion of TRPV1 channels in renal sensory nerves. This rationale was 2-fold: 1) to directly compare to a prior study using Trpv1^−/−^ rats (Stocker and Sullivan, 2023), and 2) the absence of a tool to reliably and selectively delete TRPV1 channels in renal sensory nerves. Thus, there may be confounding effects attributed to the complete knock-out that may compensate for the actions of TRPV1 channels in renal sensory nerves.

## Data Availability

The raw data supporting the conclusions of this article will be made available by the authors, without undue reservation.
